# The Link Between Physical Fitness and Cognitive Function in Vulnerable Low-Income Older Adults from Amazonas, Brazil

**DOI:** 10.3390/healthcare14020185

**Published:** 2026-01-12

**Authors:** Duarte Henriques-Neto, Alex Barreto de Lima, Miguel Peralta, Adilson Marques, Marcelo de Maio Nascimento, Andreas Ihle

**Affiliations:** 1 Research Center in Sports Sciences, Health Sciences and Human Development, University of Maia, 4475-690 Maia, Portugal; 2 Course of Physical Education, Universidade Nilton Lins, Manaus 69058-030, Brazil; alex.lima@uniniltonlins.edu.br; 3 CIPER, Faculdade de Motricidade Humana, Universidade de Lisboa, Cruz-Quebrada, 1649-004 Lisboa, Portugal; mperalta@fmh.ulisboa.pt (M.P.); amarques@fmh.ulisboa.pt (A.M.); 4 ISAMB, Faculdade de Medicina, Universidade de Lisboa, 1649-004 Lisboa, Portugal; 5 Department of Physical Education, Federal University of Vale do São Francisco, Petrolina 56304-917, Brazil; marcelo.nascimento@univasf.edu.br; 6 Swiss Center of Expertise in Life Course Research LIVES, 1227 Carouge, Switzerland; andreas.ihle@unige.ch; 7 Center for the Interdisciplinary Study of Gerontology and Vulnerability, University of Geneva, 1227 Carouge, Switzerland; 8 Department of Psychology, University of Geneva, 1227 Carouge, Switzerland

**Keywords:** cardiorespiratory fitness, cognition performance, healthy ageing, mental health, muscular fitness

## Abstract

**Background**: Studies on the association between cognitive and physical fitness in older adults from particularly vulnerable settings are scarce. This study aims to analyse the relationship between different protocols for assessing physical fitness and the cognitive function of low-income older adults. **Methods**: A total of 312 adults aged 60–96 years (M age = 72.63, SD= 7.81) living in the urban area of Amazonas, Brazil, participated in the study. The following measures of physical fitness were assessed: body composition, handgrip strength, the Five Times Sit-to-Stand Test and Senior Fitness Tests. Cognitive function was assessed using the Mini-Mental State Examination (MMSE). Linear regression models were used to analyse the relationship between physical function measures and cognitive function. **Results**: For men, only the 30-chair stand test power (β = 0.33, *p* < 0.001) presented favourable association with cognitive function. For women, significant associations between MMSE score were observed for every fitness test, except for the chair sit-and-reach test. **Conclusions**: Physical fitness is differently associated with cognitive function among low-income older men and women from Amazonas. Muscular fitness particularly seems to be an important indicator of cognitive function. It should be considered for monitoring, promoting, and managing health-ageing of low-income elderly populations of both sexes.

## 1. Introduction

The world’s population is ageing. It is expected that by 2030 around 1.4 billion people will be over the age of 60, which represents one-sixth of the global population [1]. Ageing is a continuous biological process characterized by a progressive decline in physiological reserve, which materialises in the increased incidence of health problems, such as chronic pain, decreased functionality, falls, and a cognitive and mental health decline (e.g., anxiety, depression, cognitive impairment, dementia) [1,2]. The prevalence of mental disorders in adults over the age of 60 is high [1]. Mental health determinants are multifactorial and include physiological (e.g., genetic characteristics, inflammatory processes and oxidative stress), behavioural (e.g., exercise, diet, substance use) and socio-environmental factors (e.g., educational level, living place, social support) [3,4,5,6,7,8]. These factors have a significant influence on mental health for the entire population but more so among older adults and those with fewer economic resources [9].

Physical fitness is a biomarker of health across the lifespan, as it can represent the functional capacity of different biological systems and the influence that behaviours and the environment have on them [10]. Among older adults, previous studies have linked physical fitness with metabolic health, including diabetes and cardiovascular diseases, and mental health [11,12,13].

Cardiorespiratory fitness (CRF) has emerged as a critical determinant of cognitive function [14]. Recent epidemiological investigations reinforce the neuroprotective effect of CRF on cognitive functions, demonstrating that higher CRF scores predict superior functionality in various cognitive domains and that these benefits are most evident in older adults (aged 80+) [15,16].

Global epidemiological evidence consistently indicates a significant positive association between superior performance in upper and lower-body strength assessments and cognitive performance in older adults [17,18]. Monitoring the several components of health-related physical fitness (body composition, CRF and neuromuscular fitness) can be an important strategy to anticipate and prevent such problems [19]. Similarly, monitoring cognitive function is a fundamental strategy for health systems around the world. Early identification of cognitive decline allows for a more effective intervention in the development and progression of neurodegenerative diseases, impacting mortality [20,21].

The positive association between physical fitness profile and cognitive function in older ages is well-established [22,23,24,25]. However, it remains unclear precisely which physical fitness components are most strongly associated with cognitive function [26]. Furthermore, most research contemplates samples from middle or high-income settings with a paucity of studies from particularly vulnerable settings, such as low-income populations [27]. Brazilian Amazon state presents a critical context for public health research, characterised by profound socioeconomic disparities. Approximately 20.9% of the region’s population lives below the poverty line, whilst nearly 80% lack access to basic healthcare. These structural deficits create a cycle of vulnerability for older adults residing in the interior, where logistical barriers and geographic isolation severely limit access to conventional healthcare [28]. Based on these scientific evidence gaps and theoretical rationale described above, this study aims to analyse the following hypotheses:

**Hypothesis** **1.** *Higher levels of cardiorespiratory fitness are positively associated with better cognitive performance in older adults from low-income communities in Amazonas*.

**Hypothesis** **2.** *Muscle fitness is an independent predictor of better cognitive function in low-performing older adults, even after adjusting for body composition, socioeconomic and education level*.

## 2. Materials and Methods

### 2.1. Participants and Procedures

The sample included 312 community-dwelling older adults aged 60–96 years living in the urban area of Novo Aripuanã (Amazonas, Brazil) who participated in this cross-sectional study. Novo Aripuanã (5°08′00″ S, 60°22′30″ W) is a municipality located in the interior of the Brazilian state of Amazonas [29].

**Figure 1:**
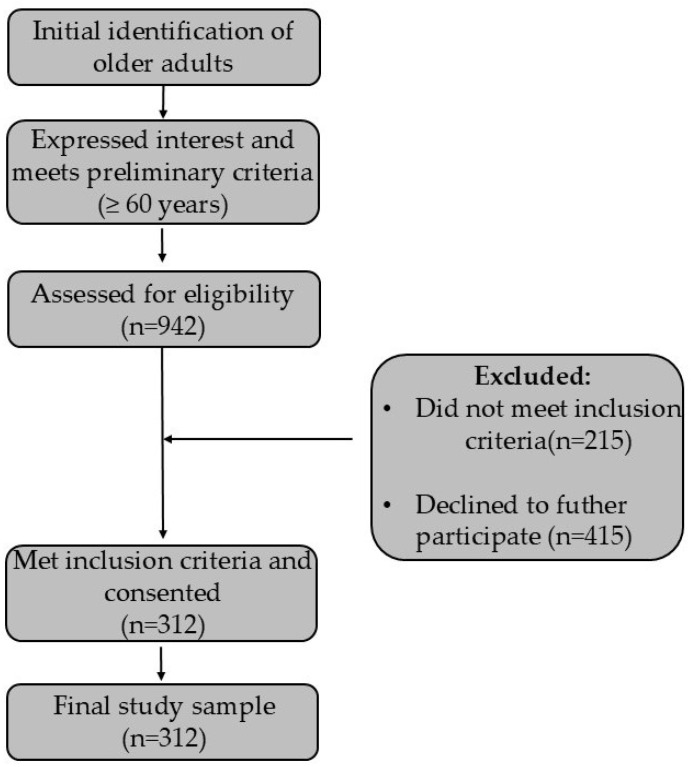
Sampling process flowchart.

Figure 1 represents the sampling process in the study.

Participants were recruited between January 2018 and January 2020 through fliers and posters in primary health units, public squares, churches, and other public places, and announcements on local radio stations. Of the 942 older adults who showed interest and met the search criteria. The following criteria were considered for participant inclusion: (1) older adults aged 60 and over, residing in the urban area of the city; (2) independent in carrying out activities of daily living; (3) having no moderate-to-severe cognitive impairment; (4) no contraindications for physical exertion; (5) without current severe joint pain or any chest pain [30]. Following screening, 630 individuals were excluded. Specific reasons for exclusion were: 178 individuals were not independent in performing activities of daily living, and 37 were excluded due to clinical information indicating contraindications for physical exertion. Furthermore, 415 individuals were excluded due to logistical constraints (lack of transportation or unavailability to attend the scheduled assessment date/location). This systematic process resulted in the final analysed sample of n = 312 participants.

#### Ethics Approval and Consent to Participate

The assessments were conducted in a single session on the premises of the Higher Studies Center of the State University of Amazonas, located in Novo Aripuanã (Amazonas, Brazil), by previously trained professionals. All participants provided written informed consent before performing the assessments and after explaining the study aim and procedures. This study was approved by the Ethics Committee of the Universidade Estudual do Amazonas, Brazil, according to the Declaration of Helsinki [31] and Resolution 466/12 of the National Health Council [32], making part of the research project: “Sarcopenic Syndrome, Physical Function, Phenotype and Quality of Life in Elderly with and without Sedentary Lifestyle” (CAAE 74055517.9.0000.5016/Referee 2.281.400). The study authors did not have access to information that could identify the participants individually during or after data collection.

### 2.2. Measures

#### 2.2.1. Cognitive Function

Cognitive function was assessed using the Mini-Mental State Examination (MMSE) [33]. The MMSE is a screening test for cognitive performance that assesses six cognitive domains, namely: orientation, repetition, attention, calculation, verbal recall, language, and visual construction. Cognitive impairment classification was determined using education-stratified cut-off scores on the specific screening instrument, ensuring robust adjustment for the documented influence of educational attainment. Specifically, the following thresholds were applied to define the presence of cognitive problems: A score < 21 was indicative of impairment for illiterate participants; a score < 22 for those with Fundamental school—I; a score < 23 for those with Fundamental school—II; and finally, a score < 24 was implemented for participants with higher school or above. This rigorous stratification process aligns diagnostic criteria with demographic variables, thereby enhancing the sensitivity and specificity of the classification across varying educational backgrounds within the study sample [34].

The version applied in this study has been validated for Brazilian older adults and is recognized as one of the most frequently used instruments for assessing cognitive status in both clinical practice and epidemiological research [25,35,36,37,38,39].

#### 2.2.2. Physical Fitness

Physical fitness assessments included the test comprising the Senior Fitness Test battery (SFT) [40], handgrip strength test, and the five-times sit-to-stand test (5x-STS).

The SFT was specially developed for adults over 60 years of age. It is primarily used to evaluate physical function in healthy elderly people but is also used for people with dementia [41]. This battery includes the protocol tests of five components: muscular strength [i.e.,: 30-s chair stand test (30-STS) and 30-s arm curl], cardiorespiratory fitness (i.e.,: 6-min walk), flexibility (i.e.,: chair sit-and-reach and back scratch), and agility/dynamic balance (i.e.,: gait speed and 8-foot up-and-go) [40,42,43].

Grip strength was assessed using a handgrip dynamometer (Camry EH10; Sensun Weighing Apparatus Group Ltd., Shenzhen, China) [44], on the dominant limb, with the participants seated, the shoulder in a neutral position, elbow flexed at 90° and the forearm in a neutral position neutral [45]. The dynamometer was adjusted according to the participant’s hand size. Throughout the measurement, they were encouraged to press the dynamometer handle with as much force as possible for five seconds [44]. The test was performed twice, with an interval of one minute, and the results were recorded in kilograms [46].

The 30-CST was performed to assess lower-body muscle strength [30] and power [47]. Participants performed as many repetitions as possible in this period. The test was performed on a standardized armless chair, 43 cm high. In a sitting position, participants kept their arms crossed over their chest. Verbal encouragement was provided during the assessment of muscle function, and participants were allowed to try twice before the definitive measurement was recorded [47].

For the 5x-STS assessment, participants were assessed in the same chair (43 cm) and physical assessment room [48]. Participants were instructed to sit with their arms crossed over their chest and their back supported on a chair. Participants were asked to stand up and sit down as quickly as possible five times. The time taken in seconds to complete the test was recorded using a stopwatch. A shorter time to perform the 5x-STS is indicative of better lower-body strength.

To ensure data validity and mitigate the potential impact of accumulated fatigue (both physical and central) on participant performance, the assessment protocol was distributed over two days. The specific testing sequence was strategically designed based on two primary physiological criteria: metabolic demand progression and biomechanical alternation. Tests with lower energy costs were prioritized before those with higher cardiorespiratory demands, with the highest expenditure test (6-min walk test) placed at the end of the protocol. Furthermore, the ordering aimed to alternate between muscle groups (upper vs. lower limbs) and physical components (flexibility vs. strength) to prevent local muscular fatigue. To ensure adequate energy substrate resynthesis and neuromuscular recovery, a standardized rest interval of at least 5 min was implemented between assessments. The tests were conducted over two days and in the following order: Day 1: (a) gait speed, (b) back stretch, (c) 5x-STS, (d) handgrip; Day 2: (a) 8-foot up-and-go, chair sit-and-reach, 30-STS, 30 s arm curl and 6-min walk.

#### 2.2.3. Body Composition

Body mass and height were measured using a calibrated mechanical anthropometric scale (110 CH, Welmy, São Paulo, Brazil), with participants barefoot, wearing light clothing, standing erect, arms hanging at their sides, heels together and occipital regions and glutes touching the vertical ruler of the scale [49]. Body mass index (BMI) was calculated as the ratio between body mass and body height squared (kg/m^2^) [50].

#### 2.2.4. Socioeconomic Status

Socioeconomic status was assessed using the questionnaire from the Brazilian Association of Research Companies [51]. The questionnaire classifies individuals into five social classes, ranging from class A (those with greater purchasing power) to class E (those with lower purchasing power) based on the possession of some consumer goods, education of the head of the family and access to public services. The method used is described in greater detail in a previous publication [51]. The cut-off criteria for each socioeconomic class were as follows: Class A (45–100 points); B (29–44 points); C (17–28 points); D and E (0–16 points) [51].

#### 2.2.5. Education Level

The educational level of participants was identified in a specific question asking participants to indicate their completed educational level at the time of the study: (0) non-literate; (1) Fundamental school—I; (2) Fundamental school—II; (3) High school; (4) Graduate or above [52]. Identification numbers at education levels were used for coding purposes in data analysis.

### 2.3. Statistical Analysis

Descriptive statistical analyses included measures of central tendency and dispersion, such as frequencies, means, and standard deviations, for all study variables. Normality was assumed under the central limit theorem, given the sufficiently large sample size, allowing the use of parametric tests for continuous variables. Comparisons between men and women were performed using Student’s *t*-tests for continuous variables and chi-square tests for categorical variables. Effect sizes were calculated using Cohen’s *d* for continuous outcomes and Cohen’s *h* for categorical proportions to provide a more informative interpretation of the magnitude of sex differences. Cohen’s criteria were adopted for interpretation: values of approximately 0.20 were considered small effects, 0.50 moderate, and ≥0.80 large for *d*; similarly, *h* values of 0.20, 0.50, and 0.80 indicated small, medium, and large effects, respectively [53]. Equality of variance was assessed using Levene’s test. Correlation analyses were performed using Pearson’s or Spearman’s coefficients according to the measurement level and distribution of each variable. Linear regression with 95% confidence intervals (CIs) was employed to examine the relationship between physical fitness and cognitive function (MMSE scores). Two models were tested: Model 1 (unadjusted) and Model 2 (adjusted for BMI, educational level, and socioeconomic status). Regression analysis using the stepwise method was conducted to investigate the incremental contribution of physical fitness, socioeconomic variables, and education level to the variance in MMSE scores. Multicollinearity was assessed using the variance inflation factor (VIF), with values below 5.0 considered indicative of no multicollinearity. Categorical variables with more than two levels were dummy coded prior to inclusion in the linear regression models, with one category designated as the reference group. Statistical significance was set at *p* < 0.05. All analyses were performed using SPSS, Version 28 (IBM Corp., Armonk, NY, USA).

## 3. Results

Demographic, cognitive and physical characteristics of the total sample (n = 312) are detailed in Table 1, with comparative analyses stratified by sex. Overall, participants had a mean age of 72.63 years and exhibited high rates of illiteracy (56.4%), with 44.2% classified as having cognitive problems based on the MMSE cut-offs. Comparative analysis between men (n = 112) and women (n = 200) revealed statistically significant differences across most physical measures, but not in demographic or cognitive variables. There were no significant sex differences in age, educational level, socioeconomic status, or the prevalence of cognitive problems (*p* = 0.123 and *p* = 0.123, respectively). Based on effect size analysis, largest and most clinically relevant sex differences were observed in physical fitness domains: handgrip strength showed the largest discrepancy (*p* < 0.001), with a large effect size (d = 1.705). Significant differences were also evident in measures of lower body power (30 s-STS: *p* < 0.001, d = 0.776), CRF (6-min Walk: *p* < 0.001, d = 0.655), and overall mobility (8-foot up-and-go: *p* < 0.001, d = 0.383). These results underscore substantial sex-based disparities in physical capacity within this population, independent of differences in cognitive status.

Bivariate analysis revealed distinctive patterns of association across biological, sociodemographic (i.e.,: education level and socioeconomic status class), and functional domains (Table 2). Sex exerted influence on functional performance, demonstrating a negative correlation with handgrip strength (r = −0.634, *p* < 0.01) and lower-limb capacity (r = −0.350 for both 30-s chair stand and 5-repetition sit-to-stand tests), indicating marked sexual dimorphism in force production. Furthermore, chronological age was significantly associated with functional decline, most notably impacting upper-limb flexibility (r = 0.456, *p* < 0.01) and muscle power. Physical parameters displayed significant correlations, with lower-limb power emerging as a key component; it showed a high magnitude of association with 30-STS power (r = 0.699) and upper-body strength (r = 0.548), whilst cognitive status displayed moderate but consistent associations with all physical fitness components, reinforcing the link between cognitive performance and physical capability.

Sex-specific unadjusted and adjusted associations between physical fitness and cognitive function in older adults from Amazonas, Brazil, presented different associations for men and women (see Table 3). Among men, lower-limb muscular power emerged as the strongest indicator of cognitive performance. In the unadjusted model, 30-s chair stand power was associated with cognitive scores (B = 0.08, 95% CI = 0.04 to 0.13, R^2^ = 0.12, *p* < 0.001), and this association remained significant after adjustment for covariates (β = 0.34, R^2^ = 0.20). The 30-STS also showed association (B = 0.37, 95% CI = 0.12 to 0.62, R^2^ = 0.07, *p* < 0.05), although the strength of the association attenuated after adjustment. No other physical fitness indicators demonstrated significant associations with cognitive scores in men.

In contrast, several fitness components were associated with cognitive performance in women. Handgrip strength showed a significant positive association both before (B = 0.20, 95% CI = 0.08 to 0.33, R^2^ = 0.05, *p* < 0.05) and after adjustment (β = 0.17, R^2^ = 0.23). Similarly, 30-STS (B = 0.08, 95% CI = 0.06 to 0.16, R^2^ = 0.08, *p* < 0.001; adjusted β = 0.20, R^2^ = 0.22) and 30-s arm curl performance (B = 0.35, 95% CI = 0.23 to 0.62, R^2^ = 0.09, *p* < 0.001; adjusted β = 0.24, R^2^ = 0.24) were consistently associated with higher cognitive performance. Additionally, better flexibility in the back scratch test, faster performance in the 8-foot up-and-go and 5-STS test, and greater distance in the 6-min walk were all significantly associated with cognitive performance in women, even after adjustment.

Regression analyses were conducted to examine the associations between physical fitness, social variables, and cognitive performance (see Table 4). Among men, only lower-limb muscular power significantly explained variance in MMSE scores. In Block 1, 30-STS power accounted for 11.5% of the variance (R^2^ = 0.115), emerging as a significant predictor (β = 0.339, *p* < 0.001). In women, the final model (Block 6) explained 28.8% of the variance in cognitive performance (R^2^ = 0.288). Educational status was a consistent determinant, with non-literate participants exhibiting lower cognitive performance across blocks (e.g., Block 6: β = –0.121, *p* = 0.004), while those with high school (β = 0.188, *p* = 0.006) and graduate-level education (β = 0.134, *p* = 0.040) showed superior cognitive performance. Physical fitness indicators contributed incrementally to the final model: the 6-min walk test (β = 0.188, *p* = 0.037), upper-body flexibility assessed via the back scratch test (β = 0.218, *p* < 0.001), and lower-limb power (30-STS; β = 0.209, *p* < 0.001) were all independently associated with higher cognitive performance. These findings indicate that, while muscular power was the sole determinant in men, cognitive performance in women was jointly influenced by educational attainment, CRF, flexibility, and muscular power.

## 4. Discussion

To the best of our knowledge, this is the first study aimed at examining the link between different physical fitness components and cognitive function among low-income community-dwelling older adults from Amazonas, Brazil, representing a particularly vulnerable population.

In line with hypotheses 1 and 2, our findings showed that physical fitness test performance was associated with cognitive performance; however, patterns of association were different for men and women.

In older women, cardiorespiratory fitness (6-min walk) played a dominant role in cognitive performance (β = 0.23, *p* < 0.01). In a setting of limited formal education, higher aerobic capacity may act as a vital physiological compensatory reserve, enhancing cortical plasticity in the prefrontal and parietal networks.

Mechanistically speculative, CRF enhances cortical plasticity, specifically by augmenting functional efficiency within the attentional networks of the prefrontal and parietal cortices, whilst concurrently mitigating hippocampal atrophy [54]. However, in men, no significant association was observed between the 6-min walk test and MMSE results, even with higher CRF results when compared to women. These findings corroborate the study of Oberlin et al., who similarly reported an association between lower educational attainment and reduced cognitive performance. Notably, their study highlighted the protective role of CRF in attenuating this adverse relationship, an effect that was particularly pronounced amongst women [16].

Our findings partially confirm hypothesis 2, indicating that muscular strength and power independently contribute to cognitive performance. Notwithstanding, the nature of this association appears to be sex specific. In men, lower-limb muscular power emerged as the only significant indicator of cognitive performance, suggesting that explosive strength rather than general endurance or flexibility may be more relevant for neurocognitive health in men. Among women, upper-limb strength assessed by the 6-min walk test, back scratch, and 30-STS power remained a significant indicator across hierarchical regression models explaining 28.8% of the variance. Notably, the indicative role of muscle strength persisted alongside other fitness domains (e.g., CRF, flexibility), reinforcing its independent contribution to cognitive performance.

Age-related decreased muscle strength and loss of skeletal muscle mass are linked to the development of cognitive impairment in later life [55]. For example, a recent prospective cohort demonstrated that more severe sarcopenia at baseline was associated with a faster rate of cognitive decline and an increased risk of developing mild cognitive impairment and Alzheimer’s dementia [56]. In line with this finding, our study also identified a positive association between muscle strength and power with cognitive function. Muscle strength was the only physical fitness component consistently associated with better cognitive function for both older men and women. Contracting skeletal muscle induces the release and up-regulation of important myokines (e.g., interleukine-6, brain-derived neurotrophic factor) dampening inflammation and oxidative burst activity and regulating synapses in the brain. Additionally, skeletal muscle contractions have immune and redox effects that modify brain function and reduce muscle catabolism [57]. On the other hand, common biomarkers of inflammation, such as interleukin-6 and tumour necrosis factor-α, have been linked with muscle weakness and age-related cognitive decline [57]. Therefore, existing evidence supports the idea that the mechanism through which muscle contractions improve cognitive function is its anti-inflammatory and neurotrophic effects, with fitter people exhibiting better profiles. Several studies have suggested that better physical fitness (e.g., gait speed, handgrip strength, chair stand test, 8-foot up-and-go test) is associated with greater cognitive performance and lower mortality independently of comorbidities [22,25,58]. The results of our study show that muscle strength is an important indicator of cognitive performance in men and women. However, whilst in women, both CRF and muscular strength are positively associated with cognitive function, in men, only lower limb muscular strength emerged as a significant predictor of better cognitive performance. These findings corroborate emerging literature suggesting that mechanisms of neuroplasticity and brain health maintenance may operate through distinct pathways depending on biological sex [59].

The discrepancy in our results reinforces the critical need to disaggregate data by sex in neuroscience and exercise physiology research. As emphasized by Kirby et al. (2024) [59], combining data from men and women can mask significant effects and reduce the sensitivity to detect sex-specific neuroplasticity mechanisms. For instance, baseline differences in myelination with evidence of higher myelin water fraction in the corpus callosum in males suggest that the neural substrate upon which physical fitness acts is structurally distinct [59]. Additionally, Luders and Kurth (2020) caution that structural differences between male and female brains are not merely a product of brain size but represent real dimorphisms that influence function and susceptibility to interventions [60].

Additionally, the results of this study identify that cognitive performance in women is multifactorial. Educational level consistently emerged as the most contributing factor, while upper limb strength, CRF, flexibility, and BMI also added explanatory power. These findings highlight the cumulative importance of cognitive reserve and physical fitness in shaping women’s cognitive trajectories, consistent with studies showing that women’s cognition is more sensitive to modifiable lifestyle factors when educational opportunities are limited [61]. Older adults often deal with multimorbidity, decreased physical and functional fitness, and social changes (i.e.,: social isolation), which promote a decrease in quality of life and an increased risk of mortality [62,63]. The lack of equal opportunities to study, social and cultural obligations (i.e., domestic tasks, motherhood, lower pay compared to men) that women are subject to are potential barriers to promoting mental health and may explain the multifactorial relationship between the variables studied and cognitive performance [64].

Our findings that lower limb muscular strength is a common denominator, yet CRF plays a crucial additional role in women, suggest that intervention strategies for cognitive decline should be personalised. While the promotion of physical activity is universally beneficial, the prescription of exercise aiming for cognitive optimisation may require different emphases: a robust focus on strength training for men and a combined approach (aerobic and strength) for women.

Promoting physical activity and exercise programs are well-known cost-effective strategies to improve health. In that context, well-structured physical activity and exercise of sufficient intensity can improve physical fitness even among older adults [65]. Furthermore, staying active can reduce stress, anxiety, and depression, all of which are linked to cognitive impairment. Thus, exercise is key to maintaining both physical and mental well-being in ageing. Existing health disparities are increased in low-income settings, such as Amazonas in Brazil. In these contexts, given the expansive benefits of exercise and its relative low-cost, public health authorities (e.g., national and regional public health agencies, public health services), decision-making institutions (e.g., municipalities and other local authorities) and key stakeholders (e.g., social partners, sport clubs, professional associations) should consider and prioritise strategies improving physical fitness (e.g., clinical and community-based exercise programs, physical activity counselling) to promote heathy ageing. Overall analysis, the findings demonstrate that both CRF and muscle strength independently contribute to cognitive performance in older adults living in low-income communities in Amazonas, although their influence varies by sex and fitness domain. Cardiorespiratory fitness appeared to be more relevant among women, whereas muscular power and strength consistently emerged as significant predictors across both sexes. These results highlight the importance of physical fitness as a determinant of cognitive health in socioeconomically disadvantaged older populations.

### 4.1. Limitations and Strengths

This study has some limitations that must be acknowledged. Firstly, the sample size cannot guarantee a representative distribution of the low-income older population from interior Amazonas State. Secondly, the cross-sectional design does not allow for the identification of predictors, so we cannot establish causality in the relationship between physical fitness performance and MMSE scores. Thirdly, a result of second limitation, it was not possible to assess the test–retest reliability of the MMSE within this specific population, preventing conclusions regarding the instrument’s reproducibility in this context. Fourth, men and women were not equally distributed, which may limit the interpretation of the values from the analyses. This also concerns the lack of evaluation of the nutritional profile, sleep quality, and more specific components of body composition (e.g., muscle mass and fat mass) and physical activity levels, which may act as confounding variables in the analyses of this research. Despite these limitations, the present study has several important strengths: The sample size is relatively large, considering the specific geographical conditions, the socio-economic conditions and especially the phenotypic profile. In addition, this research makes it possible to use physical fitness tests to monitor mental health in an epidemiological context in a population with low economic resources safely and scientifically.

### 4.2. Future Directions

Future investigations should explore whether these differences are attributable to variations in the release of exercise-induced neurotrophic factors, differences in cerebral vascularisation, or direct hormonal influences on synaptic plasticity. Additionally, we suggest that future studies use more accurate physical fitness analysis instruments (i.e., force cells and platforms, body composition analysers with more reliable variables (i.e., muscle mass, appendicular mass, phase angle), as well as instruments for assessing other components of mental health, such as symptoms of depression. Analyse a sample that is representative of the state of Amazonas and, if possible, of the whole of Brazil, to establish patterns and trends in the relationship between physical fitness and cognitive performance. Additionally, further studies with larger populations from other vulnerable settings and longitudinal designs with a multidisciplinary approach (i.e., medical doctors, exercise physiologists, psychologists) are needed.

## 5. Conclusions

This study demonstrates that physical fitness tests are differentially associated with cognitive function among low-income older men and women. While only men with greater lower-body power had a better cognitive performance, women with more lower-body strength and power, flexibility, and aerobic endurance were associated with better cognitive function. Muscular fitness may be an important indicator of cognitive function and should be considered for monitoring, promoting, and managing health-ageing of low-income elderly populations of both sexes.

## Figures and Tables

**Table 1:** Characteristics of the participants included in the study.

	Mean ± Standard Deviation OR n (%)			*t*/*x*^2^ (312)	*p*-Value	Cohen’s *d*/*h*
	All (n = 312)	Men (n = 112)	Women (n = 200)			
Age, years	72.63 ± 7.81	73.07 ± 7.31	72.39 ± 8.09	0.744	0.223	0.088
Educational level, n (%)						
Non-literate	176 (56.4)	63 (56.3)	113 (56.5)	7.252	0.123	0.152
Fundamental school—I	60 (19.2)	22 (19.6)	38 (19.0)			
Fundamental school—II	23 (7.4)	7 (6.3)	16 (8.0)			
High school	28 (9.0)	6 (5.4)	22 (11.0)			
Graduate or above	25 (8.0)	14 (12.5)	11 (5.5)			
Socioeconomic status class, n (%)						
C	18 (5.8)	5 (4.5)	13 (6.5)	0.547	0.459	0.042
D/E	294 (94.2)	107 (95.5)	187 (93.5)			
MMSE, cat (%)						
MMSE Score, pts	26.60 ± 2.46	27.09 ± 2.12	26.32 ± 2.59	1.774	0.077	0.309
Cognitive problems	138 (44.2)	49 (43.8)	89 (44.5)	0.016	0.123	0.007
No cognitive problems	174 (55.8)	63 (56.3)	111 (55.5)			
Body composition						
Body Height, cm	153.65 ± 8.22	159.99 ± 8.26	150.10 ± 5.67	11.267	<0.001	1.472
Body Mass, kg	63.70 ± 12.67	69.29 ± 11.61	60.52 ± 12.18	6.171	<0.001	0.728
BMI, kg/m^2^	26.88 ± 4.65	27.08 ± 4.64	26.76 ± 4.65	0.574	0.283	0.068
Physical Fitness						
Handgrip strength, kg	23.67 ± 9.15	31.41 ± 8.86	19.33 ± 5.87	12.931	<0.001	1.705
30 s chair stand, n	10.86 ± 3.22	11.08 ± 3.34	10.74 ± 3.15	0.908	0.187	0.107
30 s chair stand power, W	35.76 ± 16.58	43.50 ± 18.73	31.43 ± 13.47	6.004	<0.001	0.776
30-s arm curl, n	12.56 ± 3.83	13.19 ± 4.07	12.22 ± 3.66	2.161	0.019	0.255
Chair sit-and-reach, cm	4.73 ± 11.44	4.51 ± 11.18	4.85 ± 11.61	0.401	0.400	0.030
Back scratch, cm	−16.98 ± 14.72	−20.63 ± 15.34	−14.94 ± 13.98	−3.333	<0.001	0.393
8-foot up-and-go, s	8.08 ± 2.67	7.43 ± 2.06	8.44 ± 2.89	−3.246	<0.001	0.383
6-min walk, m	407.29 ± 108.43	450.76 ± 125.59	382.95 ± 88.99	5.048	<0.001	0.655
Gait speed, m/s	1.09 ± 0.36	1.20 ± 0.35	1.03 ± 0.35	−3.145	<0.001	0.371
5-time chair stand, s	12.31 ± 4.33	11.38 ± 3.88	12.83 ± 4.49	−2.870	0.002	0.339

Legend: BMI, body mass index; MMSE, Mini-Mental State Examination.

**Table 2:** Correlation analysis results for all variables.

	1	2	3	4	5	6	7	8	9	10	11	12	13	14	15	16	17	18	19	20
1-Sex	1	-	-	-	-	-	-	-	-	-	-	-	-	-	-	-	-	-	-	-
2-Age	−0.042	1	-	-	-	-	-	-	-	-	-	-	-	-	-	-	-	-	-	-
3-Non-literate	0.002	0.106	1	-	-	-	-	-	-	-	-	-	-	-	-	-	-	-	-	-
4-Fundamental school—I	−0.008	−0.014	−0.555 *	1	-	-	-	-	-	-	-	-	-	-	-	-	-	-	-	-
5-Fundamental school—II	0.032	−0.109	−0.321 **	−0.138 *	1	-	-	-	-	-	-									
6-High school	0.095	−0.047	−0.357 **	−0.153 **	−0.089	1			-	-	-	-	-	-	-	-	-	-	-	-
7-Graduate or above	−0.124 *	−0.018	−0.336 **	−0.144 *	−0.083	−0.093	1		-	-	-	-	-	-	-	-	-	-	-	-
8-Socioeconomic status class D/E	−0.042	−0.010	0.115 *	0.086	−0.035	−0.115*	−0.180 **	1	-	-	-	-	-	-	-	-	-	-	-	-
9-MMSE Score	−0.100	−0.162 **	−0.204 **	0.033	0.071	0.183 **	0.125 *	−0.215 **	1	-	-	-	-	-	-	-	-	-	-	-
10-BMI	−0.033	−0.158 **	−0.033	0.045	−0.046	−0.028	0.056	−0.029	0.132 *	1	-	-	-	-	-	-	-	-	-	-
11-Handgrip strength (kg)	−0.634 **	−0.227 **	−0.634 **	0.059	−0.047	−0.084	0.084	0.005	0.181 **	0.180 **	1	-	-	-	-	-	-	-	-	-
12-30 s chair stand (n)	−0.350 **	−0.248 **	−0.350 **	0.029	−0.038	0.047	−0.075	−0.076	0.200 **	0.129 *	0.195 *	1	-	-	-	-	-	-	-	-
13-30 s chair stand power (W)	−0.122*	−0.301 **	−0.122 *	−0.004	−0.011	0.021	0.010	−0.067	0.309 **	0.404 **	0.498 **	0.699 **	1	-	-	-	-	-	-	-
14-30 s arm curl (n)	0.014	−0.279 **	0.014	0.009	0.132*	0.038	−0.074	−0.153 **	0.267 **	0.151 **	0.338 **	0.618 **	0.548 *	1	-	-	-	-	-	-
15-Chair sit-and-reach (cm)	0.186 **	−0.136 *	0.186 **	0.031	−0.042	−0.116	−0.067	0.139 *	−0.014	−0.156 **	0.144 *	−0.001	−0.048	0.160 **	1	-	-	-	-	-
16-Back Scratch (cm)	0.181 **	0.456 **	0.181 **	−0.043	0.083	0.133 *	0.114 *	−0.170 **	0.197 **	−0.100	−0.009	0.020	−0.061	0.110	0.102	1	-	-	-	-
17-8-foot up-and-go (s)	−0.300	−0.167 **	−0.300	−0.058	−0.016	0.028	0.090	0.038	−0.235 **	−0.132 *	−0.353 **	−0.271 **	−0.311 **	−0.459 **	−0.301 **	−0.158 **	1	-	-	-
18-6-min walk (m)	0.176 **	0.260 **	0.176 **	0.130 *	−0.043	0.053	0.107	−0.132 *	0.242 **	0.018	−0.378 **	0.161 **	0.283 **	0.338 **	0.209 **	0.060	−0.344 *	1	-	-
19-Gait speed (m/s)	0.161 **	0.324 **	0.161 **	0.005	−0.059	0.030	0.025	0.104	−0.197 **	−0.133 *	−0.283 **	−0.232 **	−0.310 **	−0.287 **	0.014	0.087	0.439 **	−0.240 **	1	-
20-5-time chair stand test (s)	−0.350 **	−0.248 **	−0.350 **	−0.063	0.014	0.020	0.065	0.057	−0.165 **	−0.068	−0.329 **	−0.250 **	−0.232 **	−0.447 **	−0.205 **	−0.075	0.495 **	−0.306 **	0.306 **	1

Legend: * *p* < 0.05; ** *p* < 0.001.

**Table 3:** Associations for physical fitness with cognitive performance.

Cognitive Performance—Mini-Mental State Examination Score										
Physical Fitness	Men (n = 112)					Women (n = 200)				
	Model 1			Model 2		Model 1			Model 2	
	B	95% CI	R^2^	β	R^2^	B	95% CI	R^2^	β	R^2^
Handgrip strength (kg)	0.03	−0.06, 0.13	0.04	−0.034	0.14	0.20 *	0.08, 0.33	0.05	0.17 *	0.23
30 s chair stand (n)	0.37 *	0.12, 0.62	0.07	0.21 *	0.17	0.27 *	0.04, 0.50	0.03	0.13 *	0.21
30 s chair stand power (W)	0.08 **	0.04, 0.13	0.12	0.34 **	0.20	0.08 **	0.06, 0.16	0.08	0.20 *	0.22
30 s arm curl (n)	0.22	0.02, 0.43	0.04	0.20	0.14	0.35 **	0.23, 0.62	0.09	0.24 **	0.24
Chair sit-and-reach (cm)	0.01	0.75, −0.06	0.01	−0.03	0.13	−0.07	−0.09, 0.05	−0.01	0.02	0.18
Back Scratch (cm)	0.03	−0.03, 0.09	0.01	0.05	0.14	0.09 **	0.06, 0.16	0.25	0.24 **	0.25
8-foot up-and-go (s)	−0.36	−0.77, 0.05	0.03	−0.06	0.14	−0.44 *	−0.69, −0.20	0.06	−0.25 **	0.24
6-min walk (m)	0.01	−0.02, 0.01	0.02	−0.01	0.13	0.02 **	0.01, 0.03	0.09	0.22 **	0.24
Gait speed (m/s)	−0.31	−0,76, 4.09	0.01	−0.01	0.13	−0.51 *	−0.83, −0.17	0.09	−0.14 *	0.21
5-time chair stand test (s)	−0.01	−0.23, 0.21	0.01	0.08	0.14	−0.25 *	−0.41, −0.09	0.04	−0.20 *	0.23

Legend: Model 1 is unadjusted, and Model 2 is adjusted for age, body mass index, socioeconomic status class and education level. * *p* < 0.05; ** *p* < 0.001.

**Table 4:** Regression analysis on cognitive performance.

	B	S.E	β	t-Value	*p*-Value	R^2^
Men						
Block 1						0.115
30 s chair stand power (W)	0.082	0.020	0.339	3.778	<0.001	
Women						
Block 1						0.091
	−3.026	1.634	−0.301	−4.443	<0.001	
Block 2						0.158
Non-literate	−2.870	0.701	−0.270	−4.091	<0.001	
6-min walk (m)	0.016	0.004	0.261	3.958	<0.001	
Block 3						0.206
Non-literate	−2.449	0.670	−0.230	−3.653	<0.001	
6-min walk (m)	0.012	0.004	0.237	3.677	<0.001	
Back Scratch (cm)	0.085	0.024	0.224	3.471	<0.001	
Block 4						0.252
Non-literate	−2.449	0.670	−0.230	−3.653	<0.001	
6-min walk (m)	0.012	0.004	0.194	3.029	0.003	
Back Scratch (cm)	0.087	0.024	0.230	3.656	<0.001	
30 s chair stand power (W)	0.082	0.025	0.209	3.436	<0.001	
Block 5						0.272
Non-literate	−1.798	0.719	−0.169	−2.502	0.013	
6-min walk (m)	0.011	0.004	0.192	3.035	0.003	
Back Scratch (cm)	0.085	0.024	0.224	3.599	<0.001	
30 s chair stand power (W)	0.082	0.025	0.209	3.324	0.001	
High school	2.645	1.131	0.157	2.338	0.020	
Block 6						0.288
Non-literate	−1.284	0.755	−0.121	−1.700	0.004	
6-min walk (m)	0.011	0.004	0.188	2.991	0.037	
Back Scratch (cm)	0.083	0.023	0.218	3.538	<0.001	
30 s chair stand power (W)	0.082	0.024	0.209	3.359	<0.001	
High school	3.177	1.151	0.188	2.760	0.006	
Graduate	3.108	1.502	0.134	2.069	0.040	

## Data Availability

Data are available upon request to the corresponding author and with a plausible and scientific justification.
